# Cholera in Pregnancy: Outcomes from a Specialized Cholera Treatment Unit for Pregnant Women in Léogâne, Haiti

**DOI:** 10.1371/journal.pntd.0002368

**Published:** 2013-08-15

**Authors:** Iza Ciglenecki, Mathieu Bichet, Javier Tena, Erneau Mondesir, Mathieu Bastard, Nguyen-Toan Tran, Annick Antierens, Nelly Staderini

**Affiliations:** 1 Médecins sans Frontières, Geneva, Switzerland; 2 Médecins sans Frontières, Leogane, Haiti; 3 Epicentre, Paris, France; Massachusetts General Hospital, United States of America

## Abstract

**Background:**

The association between cholera in pregnancy and negative fetal outcome has been described since the 19^th^ century. However, there is limited published literature on the subject. We describe pregnancy outcomes from a specialized multidisciplinary hospital unit at the onset of a large cholera outbreak in Haiti in 2010 and 2011.

**Methods:**

Pregnant women with cholera were hospitalized in a specialized unit within the MSF hospital compound in Léogâne and treated using standard cholera treatment guidelines but with earlier, more intense fluid replacement. All women had intravenous access established at admission regardless of their hydration status, and all received antibiotic treatment. Data were collected on patient demographics, pregnancy and cholera status, and pregnancy outcome. In this analysis we calculated risk ratios for fetal death and performed logistic regression analysis to control for confounding factors.

**Results:**

263 pregnant women with cholera were hospitalized between December 2010 and July 2011. None died during hospitalization, 226 (86%) were discharged with a preserved pregnancy and 16 (6%) had live fullterm singleton births, of whom 2 died within the first 5 days postpartum. The remaining 21 pregnancies (8%) resulted in intrauterine fetal death. The risk of fetal death was associated with factors reflecting severity of the cholera episode: after adjusting for confounding factors, the strongest risk factor for fetal death was severe maternal dehydration (adjusted risk ratio for severe vs. mild dehydration was 9.4, 95% CI 2.5–35.3, p = 0.005), followed by severe vomiting (adjusted risk ratio 5.1, 95% 1.1–23.8, p = 0.041).

**Conclusion:**

This is the largest cohort of pregnant women with cholera described to date. The main risk factor identified for fetal death was severity of dehydration. Our experience suggests that establishing specialized multidisciplinary units which facilitate close follow-up of both pregnancy and dehydration status due to cholera could be beneficial for patients, especially in large epidemics.

## Author Summary

Cholera in pregnancy has been long associated with high rates of stillbirths and abortions, but there is very limited published literature describing this association or possible mechanisms. During the major cholera epidemic that hit Haiti in October 2010, we set-up a specialized cholera treatment unit for pregnant women inside the Médecins sans Frontières hospital in Léogâne, allowing for intensive follow-up of cholera-associated dehydration and of pregnancy, and facilitating access to high-quality obstetric and neonatal services in case of complications. To describe the pregnancy outcomes and risk factors for fetal death, we analyzed routinely collected data from patient files. Of 263 women hospitalized, 21 (8%) lost their pregnancy during hospitalization for cholera; an additional 16 (6%) delivered a live baby at the hospital, and the remaining 226 women (86%) were discharged with preserved pregnancy. The risk factor most strongly associated with fetal demise was severity of dehydration at admission. In large epidemics, multidisciplinary units can help prevent negative maternal, fetal and neonatal outcomes.

## Introduction

The association between cholera in pregnancy and negative fetal outcome has been described since the 19^th^ century [1]. Modern published literature reports fetal loss rates during cholera episodes as varying between 2% and 36% [2]–[7]. However, comparison of outcomes among different reports is difficult, due to differences in inclusion criteria (trimester of pregnancy, severity of dehydration, hospitalization, and biological confirmation), treatment provided, and the development level of countries where these reports originated. Although the exact cause of fetal death during a cholera episode has not been identified, several studies suggest an association between fetal loss and the degree of dehydration and hypovolemia [3], [5]–[7]. Correction of hypovolemia is further complicated in pregnancy, since it can be difficult to accurately estimate the degree of dehydration, especially during the second half due to maternal physiological hypervolemia [8].

In Haiti, over 500,000 cholera cases and 7,000 deaths were registered between the beginning of the epidemic in October 2010 and early 2012 [9]. Since the outbreak began, anecdotal accounts from treatment units suggested high fetal loss among women delivering in cholera treatment units; for example, during the first weeks of the outbreak, Médecins Sans Frontières (MSF) reported 14 stillbirths among the 17 deliveries at one of its cholera isolation units in an obstetric hospital in Port-au-Prince [10]. The objective of this retrospective study was to describe pregnancy outcomes and identify risk factors for negative outcome from routinely collected data in a specialized cholera treatment unit within an MSF hospital in Léogâne.

## Methods

### Setting

Léogâne, a town of about 80,000 inhabitants situated 50 km west of Port-au-Prince, was almost completely destroyed during the 2010 earthquake. In November 2010, MSF set up a cholera treatment center in the outskirts of the town, as well as a specialized isolation unit for pregnant cholera patients inside the hospital compound. The hospital, a second level district facility, serves the population of the town and the commune of Léogâne (300,000 people) and includes an obstetric and gynecology department with a neonatal intensive care unit. The obstetric department is particularly busy, averaging 300 deliveries per month in 2010.

The cholera isolation unit for pregnant women, with a capacity of 20 beds, was established within the hospital compound. Following standard isolation procedures, the unit was separated by a fence from the rest of the hospital and had independent water and sanitation facilities. The unit comprised a delivery room and, in a separate container building, an operating theatre for emergency surgical obstetric interventions.

### Patients and admission criteria

The isolation unit was open to all pregnant women with suspected cholera. Pregnancy status was by self report. A suspected cholera case was defined as any patient presenting with three or more liquid stools and/or vomiting episodes in the previous 24 hours. Association of pregnancy with cholera was classified as a complication, and therefore hospitalization was offered to all pregnant women with cholera, regardless of their level of dehydration. Other cholera treatment centers in the region were advised to refer pregnant women with suspected cholera to this unit.

### Cholera treatment

Patients' dehydration status was classified according to World Health Organisation (WHO) categories: no dehydration (plan A), moderate dehydration (plan B) or severe dehydration (plan C) [11]. WHO and MSF guidelines for cholera treatment were followed [11], [12]. In summary: patients with severe dehydration (plan C) received 100 ml of Ringer's lactate IV per kg of body weight in the first 3–4 hours (30 ml/kg in the first hour, and 70 ml/kg in next 3 hours), patients with moderate dehydration (plan B) received 75 ml/kg of Ringer's lactate IV in the first 4 hours, and patients without signs of dehydration (plan A) received maintenance fluids only (2 l of IV Ringer lactate per day). To correct on-going losses, all patients received 250 ml of ORS orally (or Ringer lactate intravenously in case of vomiting) after each stool. Once the dehydration was corrected, patients continued to receive IV maintenance fluids, with continuous correction of ongoing losses.

As for any cholera patient, the objective was to rehydrate the patient rapidly, but we also wanted to avoid any dehydration episodes that might not be dangerous for the mother but could cause hypoperfusion of the placenta leading to fetal death. We privileged intravenous fluid replacement because pregnant women are more likely to experience nausea and vomiting and therefore have difficulty drinking enough fluids to replace the losses immediately.

Throughout their hospitalization the dehydration status of each patient was closely monitored clinically, including the measurement of blood pressure and pulse rate. No laboratory tests were done systematically, but blood glucose level was measured in cases where hypoglycemia was suspected.

During the early phases of Haiti's cholera epidemic, we observed several cases of severe hypoglycemia in adults. As a preventive measure, all pregnant women in our cholera unit therefore received a 50 ml bolus of 50% glucose upon admission. Patients who were vomiting or unable to drink ORS received an additional 50 ml of 50% glucose in each liter of Ringer's lactate. The level of plasma glucose was monitored and replacement adjusted accordingly.

All patients received oral antibiotics (erythromycin), in order to reduce the purging rate, shorten its duration and reduce the excretion of *Vibrio cholerae* in the stool [13].

### Obstetrical care

Patients in their second or third trimester were encouraged to lie in the left decubitus position, to prevent compression of the inferior vena cava.

Fetal status was closely monitored by a midwife through the presence of fetal movement and heartbeat (confirmed by fetal Doppler). In the absence of fetal heartbeat, fetal status was checked by ultrasonography. Tocolysis was available in case of premature labor.

Standard delivery procedures were followed, and the perineal region and newborn baby were disinfected with 0.05% chlorine solution. In cases of neonatal complications, immediate resuscitation took place in the delivery room and referral to the intensive care neonatal unit was done as needed. Newborns without severe complications remained hospitalized with their mothers until discharge. Exclusive breastfeeding was encouraged and maternal breasts were disinfected with 0.05% chlorine solution before each feeding.

Cases of incomplete abortion were treated according to MSF protocol [14]. Cases of intrauterine fetal death were confirmed by ultrasonography and managed with misoprostol, after stabilization of cholera symptoms.

### Discharge criteria

Patients remained hospitalized until they fully recovered from cholera and completed treatment for any obstetrical complications. Before discharge, all patients received a cholera health education session.

### Data collection

The unit adapted the standard cholera patient file and, upon admission, recorded demographic data (age) and information related to pregnancy (gestational age by week, parity and gravidity), cholera episode (estimation of dehydration status, blood pressure, pulse rate, temperature at admission, time of onset), and fetal status (presence of fetal heartbeat, movement). Dehydration level was classified as described above [11]. Patients were closely monitored during hospitalization and relevant surveillance data was recorded, including dehydration status, number of stool and vomiting episodes, amount of fluid and any medication received, and fetal status monitoring. Relevant events during hospitalization, such as miscarriage, delivery and procedures undertaken, were also reported. At discharge, the outcome of the patient and the pregnancy was recorded.

### Data entry and analysis

Data was entered in Excel and analyzed using Stata 9.0 statistical package (College Station, Texas, USA). Intrauterine fetal death was defined as fetal death at any time during pregnancy. Risk ratios for fetal death were calculated; to control for confounding factors, variables from univariate analysis (significant at p<0.4 level) were included into the logistic regression model, using backward selection (likelihood ratio test, p<0.05). Missing data were not imputed.

### Ethical approval

The analysis was based on routinely collected clinical data from an MSF program, conducted in agreement with the Ministry of Health in Haiti; therefore ethical review and individual patient consent were not sought. Data used for analysis were anonymized.

## Results

Between 13 December 2010 and 18 July 2011, 264 pregnant women with cholera were hospitalized in the isolation unit. This analysis was performed on records from 263 patients, with one excluded because the outcome of the pregnancy was not reported.

**Figure 1:**
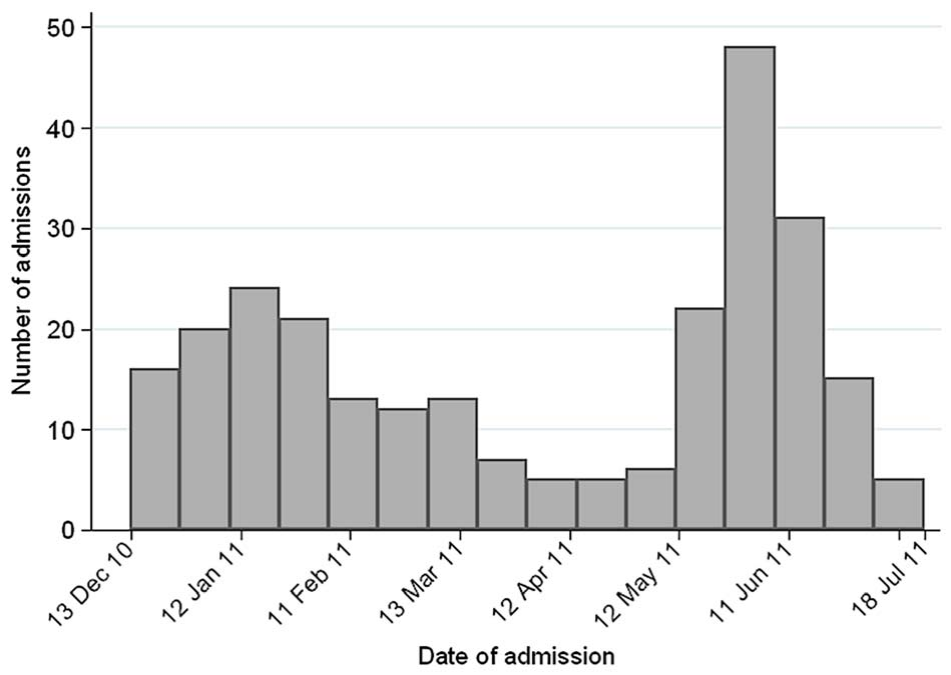
Number of pregnant women admitted in the cholera treatment unit per week. Léogâne, Haiti, 2010–2011.

The number of patient admissions varied from less than 10 per week in mid-April 2011 to almost 50 in the first week of June 2011 (Figure 1). This trend follows the seasonal pattern of suspected cholera cases reported in 2011, which peaked at the beginning of the rainy season in early June [9].

### Patient characteristics

The median age was 26 years (range 16–43 years). Half of all patients were in their second trimester of pregnancy, 34% were in the third, and the remaining 14% in the first trimester. For 22% of patients it was their first pregnancy, for 44%, their second or third one, and for the remaining 33%, their fourth or more (up to 14 pregnancies).

Among 263 women in this analysis, 14 had obstetric complications at admission: these included preeclampsia (3), urinary tract infection (3), vaginal bleeding (3), hypertension with kidney failure (1), vaginal infection (1), pre-existing severe vomiting (1), jaundice (1), and ruptured membranes (1). Two patients were febrile (axillary temperature >38°C) at admission. Five women arrived with threatened pre-term labor and were given tocolysis.

The median delay in seeking treatment was 1 day (range 0–8 days). On admission 51% of patients were not dehydrated, 42% were moderately dehydrated, and 6% were severely dehydrated; almost all patients passed watery stools. The median number of stools per cholera episode was 34 (range 0–311). Almost 60% of patients were vomiting during hospitalization. Patients received on average 12 litres of Ringer's lactate. The median length of stay was 3 days (range 0–10 days).

### Outcomes

No pregnant women died during hospitalization. No signs of fluid overload were reported.

Of the 263 hospitalized pregnant women in this analysis, 226 (86%) were discharged with a preserved pregnancy; 16 (6%) women delivered a live baby of 35–40 weeks of gestation. 21 pregnancies (8%) resulted in intrauterine fetal death (Table 1) .

**Table 1:** Pregnancy outcomes in specialized cholera unit, Léogâne, Haiti, December 2010–July 2011.

Outcome	
Preserved pregnancy	226 (86%)
Fetal death	21 (8%)
Live birth	16 (6%)

Among these 21 intrauterine fetal deaths, 10 occurred before the mother's arrival at the cholera treatment unit and 9 occurred after admission; timing of the remaining 2 fetal deaths could not be determined. Of the 9 fetal deaths that occurred during hospitalization, the median delay between admission and time that fetal death was noticed or confirmed was 48 hours (mean 62 hours, range 7–144 hours). There was no difference in dehydration status of the women whose fetuses died before or after admission. For 7 patients the dehydration level was recorded at the time confirmation of fetal death: 1 was severely dehydrated, 5 were moderately dehydrated and 1 was not dehydrated. Nearly all fetal deaths occurred during the second (10/21) or third trimester (8/21) of pregnancy.

Among the 16 singleton newborns there were 2 neonatal deaths. Both were low birth weight (<2500 g), one born at 35 weeks of gestation and the other at 40 weeks. One death occurred on day 5 postpartum and was associated with congenital malformations and necrotic enteritis. The second death occurred after 24 hours postpartum in a newborn with an Apgar score of 2 and who showed no improvement. Both newborns were admitted to the hospital's intensive care. For the remaining 14 newborns, we were able to contact 9 mothers at 1 to 5 weeks after their discharge from the treatment unit. All children were alive and healthy at the time of our call, apart from one infant who was sick with flu-like syndrome.

### Pregnancy outcomes and risk factors

The risk factors associated with fetal death during cholera in pregnancy are shown in Table 2 and were linked to the severity of the cholera episode: dehydration status on admission, number of stools passed, presence and number of vomiting episodes, number of litres of Ringer's lactate received during the treatment, and length of hospitalization. Risk factors for complicated pregnancy and childbirth, such as young or older age and the number of previous pregnancies and deliveries were not associated with negative outcome during cholera in pregnancy.

**Table 2:** Risk factors for fetal death during cholera episode. Léogâne, Haiti, December 2010–July 2011.

	Positive outcome	Fetal death	RR*	RR adjusted
	N = 242	N = 21	(95% CI†)	(95% CI)
**Age**				
>20	21 (9%)	4 (19%)	1	
20–35	184 (76%)	15 (71%)	0.56 (0.26–1.23)	
>35	35 (14%)	2 (10%)	0.45 (0.14–1.43)	
Missing	2 (1%)			
**Trimester**				
First	35 (14%)	3 (14%)	1	
Second	124 (51%)	10 (48%)	0.94 (0.27–3.26)	
Third	82 (34%)	8 (38%)	1.12 (0.31–4.02)	
Missing	1 (0.4%)			
**Gravida**				
First	51 (21%)	7 (33%)	1	
2–3	108 (45%)	8 (38%)	0.57 (0.22–1.50)	
>4	82 (34%)	6 (29%)	0.56 (0.20–1.60)	
Missing	1 (0.4%)			
**Parita**				
0	57 (26%)	7 (33%)	1	
1–2	114 (47%)	9 (43%)	0.67 (0.26–1.71)	
>3	70 (29%)	5 (24%)	0.61 (0.20–1.82)	
Missing	1 (0.4%			
**Delay to treatment**				
< = 24 hours	84 (35%)	9 (43%)	1	
>24 hours	151 (62%)	11 (52%)	0.70 (0.30–1.63)	
Missing	7 (3%)	1 (5%)		
**Dehydration at admission**				
None	132 (55%)	4 (19%)	1	1
Moderate	99 (41%)	11 (52%)	3.4 (1.11–10.38)	2.76 (0.77–9.98)
Severe	10 (4%)	6 (29%)	12.75 (4.01–40.44)*	9.44 (2.50–35.28)*
Missing	1 (0.4%)			
**Vomiting (** # ** of episodes)**				
No	98 (41%)	2 (10%)	1	1
1–10	94 (39%)	10 (47%)	4.81 (1.08–21.40)*	3.16 (0.70–14.26)
>10	31 (13%)	7 (33%)	9.21 (2.00–42.38)*	5.05 (1.07–23.83)*
Missing	19 (8%)	2 (10%)		
**Stools (** # ** of episodes)**				
0–30	100 (41%)	2 (10%)	1	
>30	125 (52%)	17 (81%)	6.10 (1.44–25.84)*	
Missing	17 (7%)	2 10%)		
**Length of stay (days)**				
<2	58 (24%)	1 (5%)	1	
2–4	123 (51%)	5 (24%)	2.30 (0.27–19.29)	
>5	40 (17%)	13 (62%)	14.47 (1.95–106.91)*	
Missing	21 (9%)	2 (10%)		
**Ringer lactate (liters)**				
0–10	136 (56%)	4 (19%)	1	
>10	94 (39%)	16 (76%)	5.09 (1.75–14.79)*	
Missing	12 (5%)	1 (5%)		

* RR, risk ratio.

† CI, confidence interval.

# , P-value<0.05.

After adjusting for confounding factors, the severity of dehydration remains the strongest risk factor for fetal death during cholera in pregnancy (adjusted risk ratio (RR) for severe vs. mild dehydration is 9.4 (95% CI 2.5–35.3, p = 0.005)). Severe vomiting also remains a statistically significant risk factor, independent of the severity of dehydration (RR 5.1, 95% 1.1–23.8, p = 0.041). The severity of dehydration remains the most important risk factor once fetal deaths that occurred before the admission are excluded from the analysis.

## Discussion

No woman in this cohort died of cholera or obstetric complications, but 21 fetal deaths (8% of all pregnancies) were recorded. 16 women (6% of all admissions) had a normal singleton delivery during her cholera episode, with 2 reported as neonatal deaths. These results are within the proportions of intrauterine fetal deaths documented in the published literature on cholera in pregnancy, ranging from 6% in Peru in 1991 to 33% in India in 1968. A simple aggregation of data gives a risk of 18% [2]–[7]. However, these results are difficult to compare, as the design of the studies and their inclusion criteria differed greatly. Some of the studies were conducted before the standardized WHO classification of dehydration status was used, and before the introduction of ORS and standardized treatment protocols. In the few articles published after the standardization of the dehydration classification and treatment protocols, the risk of fetal death was found to be 6% in 2 different studies in Peru in 1991 [5], [6] and 12% in Senegal in 2006 [7].

In this study, the risk of intrauterine fetal death was nine times higher in patients with severe dehydration compared to those with only mild dehydration. In addition, women whose dehydration status was recorded at the time when fetal death was first noticed were all, with one exception, still clinically dehydrated. All studies describing the degree of dehydration or hypovolemia demonstrated an association between the degree of dehydration and fetal loss [3], [5]–[7]. This association may be explained by the possible mechanism where severe maternal dehydration leads to critical hypovolemia, which compromises placental and fetal perfusion and results in severe fetal hypoxia and acidosis (eventually leading to fetal death). Alternatively, the gastrointestinal loss of bicarbonate could directly contribute to maternal acidosis [3]. Severe maternal acidosis also appears to be implicated in compromising fetal survival in pregnant women with diabetes type 1, as shown by the high fetal mortality ratio of 35% in pregnant women with ketoacidosis [15].

Estimating the degree of dehydration in pregnancy is difficult, in particular towards the end of pregnancy due to the increase in plasma volume: a pregnant woman can lose as much as 30–35% of blood volume during acute bleeding without changes in clinical status [8]. Although the loss of volume through diarrhea and vomiting is different from bleeding, underestimation of the degree of dehydration in pregnancy is likely. For these reasons, treatment of these patients included earlier and more intense fluid replacement to avoid hypovolemic episodes. While this could potentially lead to complications due to fluid overload, especially in case of severe maternal anemia, we did not see these complications in our patient cohort. However, close monitoring of rehydration among anemic women is of particular importance, especially in regions with high malaria prevalence.

Severe vomiting was another risk factor for fetal death, independent of the severity of dehydration. Changes in electrolytes in the amniotic fluid of pregnant women suffering from cholera have been described in the literature [16], but it remains unclear how these affect the fetus.

To mitigate the potential effects of severe vomiting on the fetus patients were routinely given glucose at admission, based on empirical observation of several severe hypoglycaemia cases in adults (usually seen only in children with cholera) during early stages of the outbreak in Haiti. Systematic measurement of glucose level at admission, followed by management (if needed) based on laboratory findings, might be more appropriate.

Finally, cholera toxin seems unlikely to play an important role in fetal death since it is not-absorbed, although it is the primary driver of the secretory diarrhoea that leads to the severe maternal dehydration associated with fetal demise.

A limitation of these results is that no suspected cholera cases were confirmed by a laboratory. While it is acceptable not to have routine laboratory confirmation of cholera cases once an epidemic has been declared, it is likely that some patients who fit the cholera case definition did not have cholera but, instead, another diarrheal disease or possibly gastrointestinal disorders associated with pregnancy. Pregnancy was ascertained only by self-reporting by women, and it is likely that first trimester pregnancies are under-represented in this sample. We did not collect information about several additional risk factors that could also influence pregnancy outcome, such as antenatal care attendance, complications and outcomes of previous pregnancies, or maternal nutritional status and anemia. Since this analysis used routinely collected data, the quality of collection was not supervised, and there were a number of missing values.

The major limitation of this analysis is that there was no control group, so we cannot readily assess the impact of our adapted treatment protocol or of closer clinical monitoring of cholera and pregnancy. However, specialized units can play an important role since they allow a multidisciplinary and focused approach. In most settings, pregnant women with cholera are usually hospitalized together with other cholera patients; delivery or miscarriage cases are either handled inside a cholera unit by staff that are often not qualified in women's health, or transferred to the maternity department, which is then put at risk of cholera contamination. In a specialized unit, conditions related to both cholera and pregnancy can be monitored simultaneously, allowing timely detection and management of obstetric and neonatal complications by qualified staff. Specialized units can also provide more privacy and dignity, especially in case of delivery or miscarriage. Such units might only be feasible in large epidemics in urban settings. But being aware of particular risk group among pregnant cholera patients can help health workers to better prepare for possible complications, such as in advance identifying possibility of referral in case of obstetric complications or availability of the basic material for conducting safe delivery.

The ideal way to prevent fetal deaths due to cholera in pregnancy would be to prevent cholera. Currently available oral cholera vaccines are not contraindicated in pregnancy [17] and have been recently shown to be safe [18]. WHO suggests that pregnant women as an especially vulnerable group might be specifically targeted for vaccination in endemic areas [17].

### Conclusion

We describe pregnancy outcomes of cholera patients during a cholera epidemic in Haiti. Our findings are in line with the hypotheses of previous authors, suggesting a link between the risk of fetal death and the severity of maternal dehydration due to cholera.

Close supervision of the hydration status of pregnant women, as well as availability of high quality obstetric and neonatal services, can help prevent negative maternal, fetal and neonatal outcomes. In addition, this strategy reinforces a woman-centered approach to patient care and helps protect her dignity. Our experience in Haiti suggests that setting up specialized multidisciplinary units to treat pregnant women with cholera could be beneficial, especially in large epidemics. Further research is needed to better understand the mechanism of fetal death during cholera episode, to estimate the degree of dehydration in pregnancy, using laboratory tests or others, and to propose better adapted treatment protocols for this high risk group of cholera patients. While clinical trials for such relatively rare events might be difficult to conduct, we encourage other clinicians treating cholera patients to document the pregnancy outcomes and any adaptation of treatment protocols, which might contribute to the understanding and treatment of these neglected patients.

## Supporting Information

Checklist S1 STROBE checklist.
(DOC)
